# Supplementary material for: Differential hepatic distribution of insulin receptor substrates causes selective insulin resistance in diabetes and obesity
Source: Nat Commun. 2016 Oct 6;7:12977. doi: 10.1038/ncomms12977 (PMC5059684; doi:10.1038/ncomms12977)
Supplement: Supplementary Information — Supplementary Figures 1-8. [file ncomms12977-s1.pdf]

Supplementary Figure 1

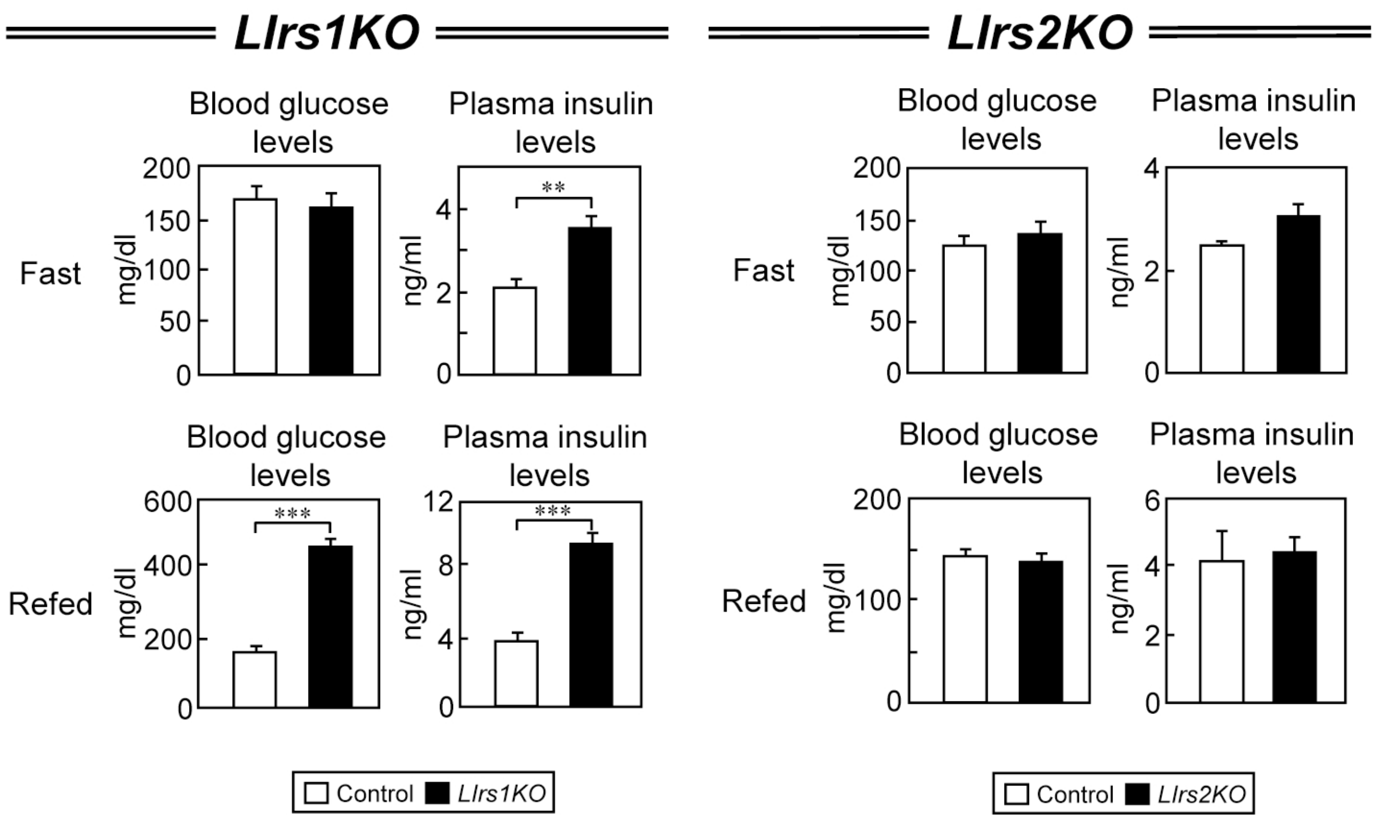

Supplementary Figure 1.

Blood glucose and plasma insulin levels in the *LIrs1KO* and *LIrs2KO* mice

Blood glucose and plasma insulin levels in the *LIrs1KO* and *LIrs2KO* mice under the fasting and fed conditions (n = 5-10).

Results are expressed as mean ± s.e.m. $^{**}P < 0.01$, $^{***}P < 0.001$.

Supplementary Figure 2

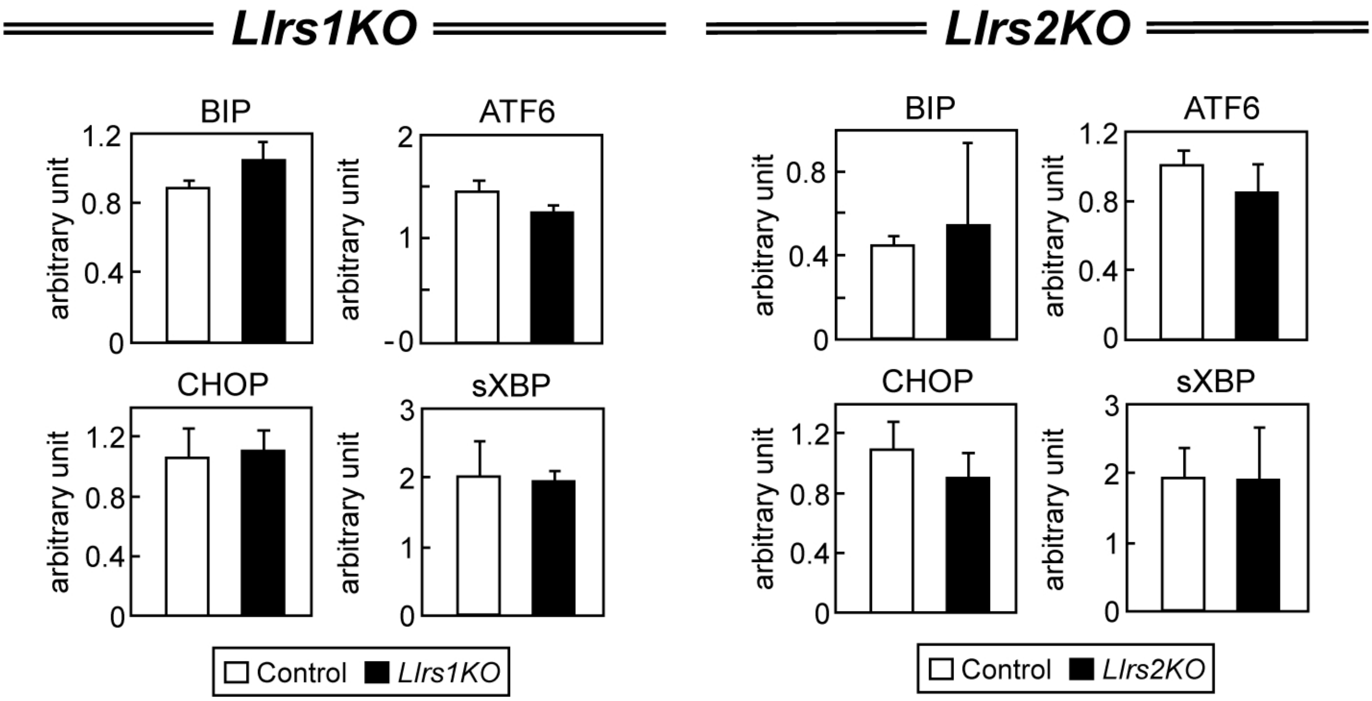

Supplementary Figure 2.

The expressions of BIP, CHOP, ATF6 and sXBP were not significantly different between the control and *LIrs1KO* mice or control and *LIrs2KO* mice.

Expression levels of the ER stress marker genes in the the *LIrs1KO* and *LIrs2KO* mice (n = 10-12). Results are expressed as mean ± s.e.m.

Supplementary Figure 3

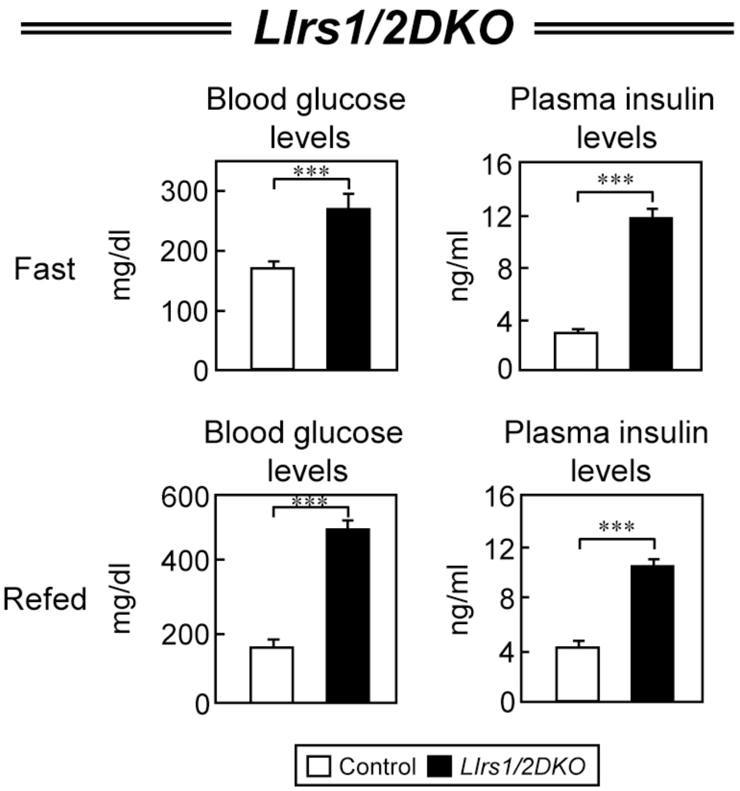

Supplementary Figure 3.

Blood glucose and plasma insulin levels were increased in the *LIrs1/2DKO* mice

Blood glucose and plasma insulin levels in the *LIrs1/2DKO* mice under the fasting and fed conditions (n = 5-12).

Results are expressed as mean ± s.e.m. ***$P < 0.001$.

Supplementary Figure 4

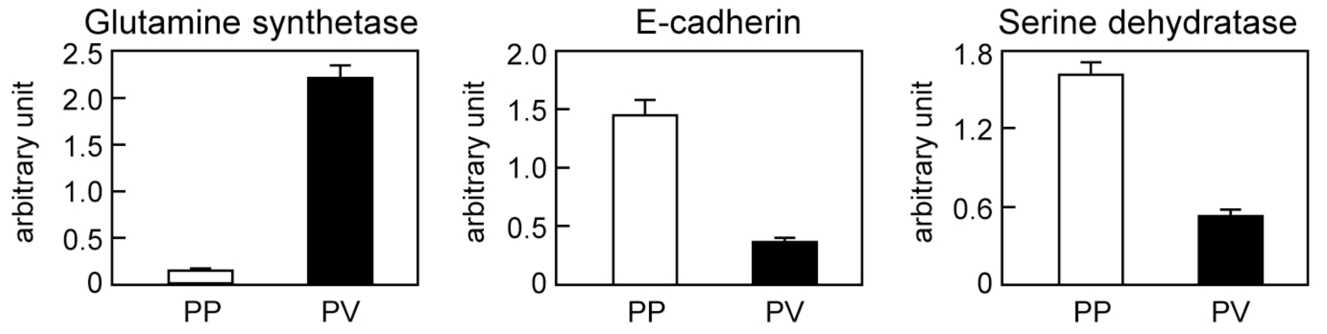

Supplementary Figure 4.

Expression levels of glutamine synthetase, E-cadherin and serine dehydratase in the hepatic PP and PV zones

Real-time RT-PCR analysis to determine the expression levels of glutamine synthetase, E-cadherin and serine dehydratase in the hepatocytes of the PP and PV zones in the livers obtained from mice reared on NC diet (n = 6-8).

Results are expressed as mean ± s.e.m.

Supplementary Figure 5

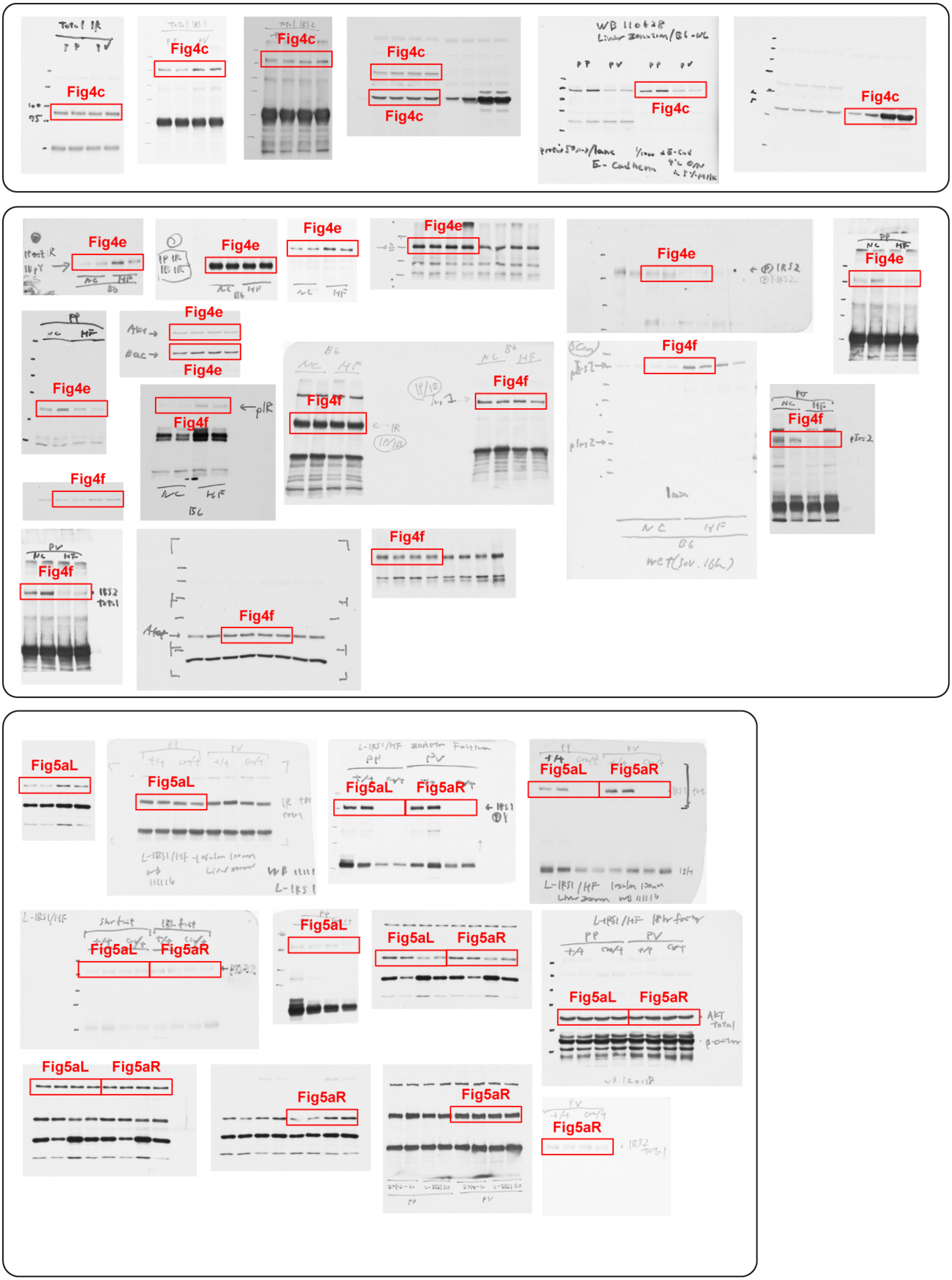

Supplementary Figure 5.

Uncropped Western blots related to Figure 4, 5

Supplementary Figure 6

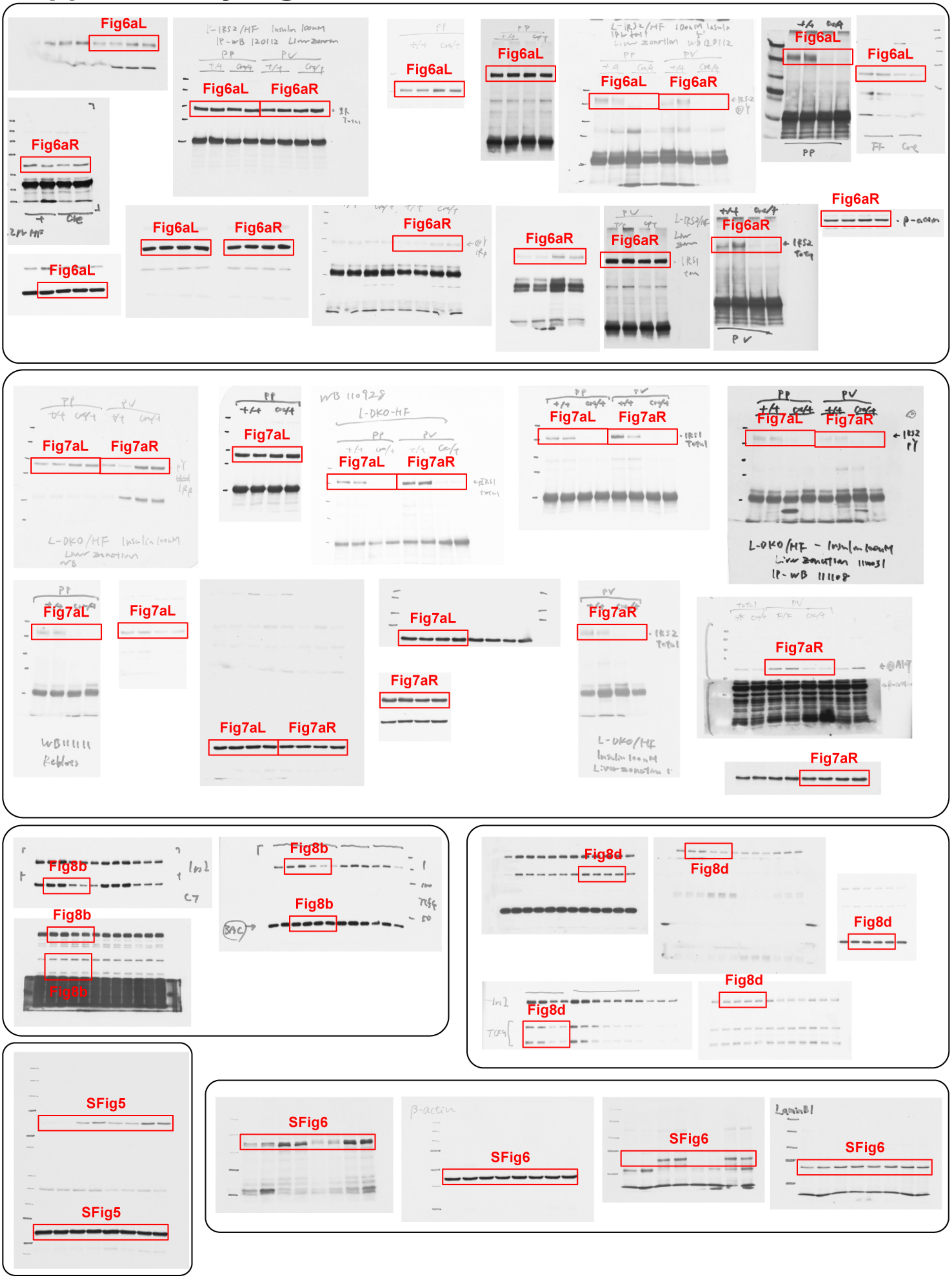

Supplementary Figure 6.
Uncropped Western blots related to Figure 6, 7, 8 and Supplementary Figure 5, 6

Supplementary Figure 7

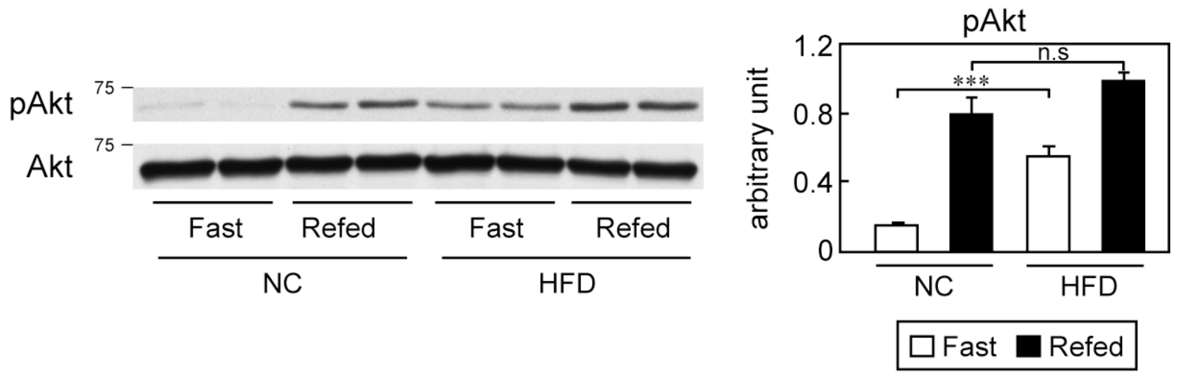

Supplementary Figure 7.

Phosphorylation levels of Akt in the fasting state were significantly increased in the mice on a HF diet as compared to those on NC diet

Phosphorylation levels of Akt in the livers of the NC and HF diet-fed mice under the fasting and fed conditions (n = 4).

Results are expressed as mean ± s.e.m. **P* < 0.05, ****P* < 0.001.

Supplementary Figure 8

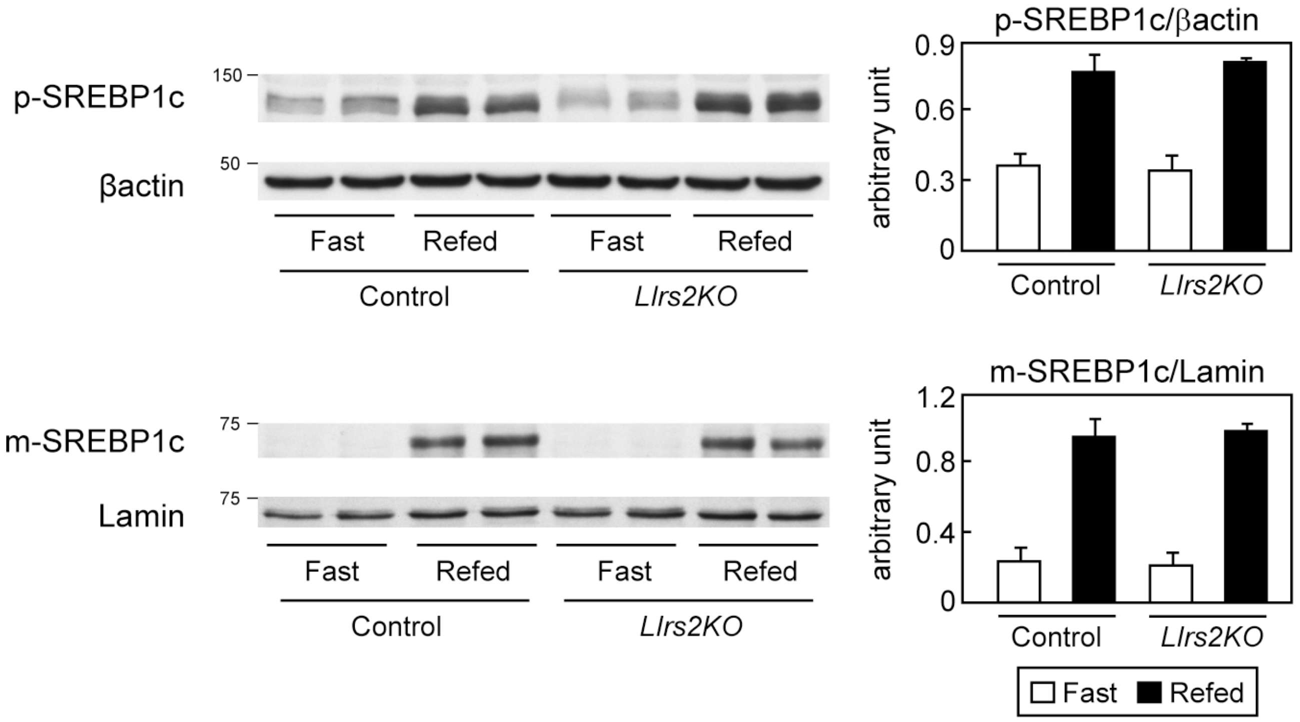

Supplementary Figure 8.

Expression levels of the precursor and mature forms of SREBP1c protein did not differ between the control and *LIrs2KO* mice

Expression levels of the precursor and mature forms of SREBP1c protein in the *LIrs2KO* mice under the fasting and fed conditions (n = 4).

Results are expressed as mean ± s.e.m.
